# Conditional Knockout in Mice Reveals the Critical Roles of *Ppp2ca* in Epidermis Development

**DOI:** 10.3390/ijms17050756

**Published:** 2016-05-18

**Authors:** Chao Fang, Lei Li, Jianmin Li

**Affiliations:** 1 Department of Pharmacology, School of Basic Medical Sciences, Nanjing Medical University, Nanjing 210029, China; fangchaonjmu@hotmail.com; 2 Translational Medicine Center, Yancheng No. 1 People’s Hospital, Yancheng 224000, China; leilipaper@outlook.com; 3 Model Animal Research Center of Nanjing Medical University, Nanjing 210029, China; 4 Key Laboratory of National Reproductive Medicine Department of Cell Biology and Medical Genetics, Nanjing Medical University, Nanjing 210029, China; 5 Collaborative Innovation Center for Cardiovascular Disease Translational Medicine, Nanjing Medical University Nanjing, Nanjing 210029, China; 6 Department of cell biology, School of Basic Medical Sciences, Nanjing Medical University, Nanjing 210029, China

**Keywords:** *Ppp2ca*, hair loss, *Krt14-Cre*, conditional knockout

## Abstract

The epidermis is an important tissue in *Homo sapines* and other animals, and an abnormal epidermis will cause many diseases. Phosphatase 2A (PP2A) is an important serine and threonine phosphatase. The α isoform of the PP2A catalytic subunit (*Ppp2ca* gene encoding PP2Acα) is critical for cell proliferation, growth, metabolism and tumorigenesis. However, to date, no study has revealed its roles in epidermis development. To specifically investigate the roles of PP2Acα in epidermis development, we first generated *Ppp2ca^flox/flox^* transgenic mice, and conditionally knocked out *Ppp2ca* in the epidermis driven by *Krt14-Cre*. Our study showed that *Ppp2ca^flox/flox^*; *Krt14-Cre* mice had significant hair loss. In addition, histological analyses showed that the morphogenesis and hair regeneration cycle of hair follicles were disrupted in these mice. Moreover, *Ppp2ca^flox/flox^*; *Krt14-Cre* mice had smaller size, melanin deposition and hyperproliferation at the base of the claws. Accordingly, our study demonstrates that PP2Acα plays important roles in both hair follicle and epidermis development. Additionally, the *Ppp2ca^flox/flox^* mice generated in this study can serve as a useful transgene model to study the roles of PP2Acα in other developmental processes and diseases.

## 1. Introduction

The epidermis of mammalian skin forms a barrier that plays key functions in the regulation of the body temperature and in protecting animals against dehydration, mechanical stress and infections [1,2]. An abnormal epidermis will lead to many diseases [3]. Phosphatase 2A (PP2A) is an important serine and threonine phosphatase that is involved in cellular activities such as cell metabolism, DNA replication, cell cycle, and cell differentiation. Several studies have found links between PP2A and many diseases [4,5,6]. The PP2A heterotrimeric holoenzyme composed of three subunits which, respectively, are structural subunit (A), regulatory subunit (B) and catalytic subunit (C). The catalytic subunit of PP2A has two isoforms, PP2Acα and PP2Acβ, whose amino acid sequences are very similar; nevertheless, the expression of PP2Acα is 10 times higher than that of PP2Acβ [7]. *Ppp2ca* plays a significant role in human diseases, such as neurodegenerative diseases, cancer, and diabetes, and its loss in mice causes early embryonic lethality at day E6.5 [8,9]. The effect of PP2Acα on the morphogenesis and regeneration of hair follicles remains unclear.

This study was conducted with the objective of studying the effects of PP2Acα on postnatal morphogenesis and homeostasis in the epidermis. To this end, we generated a *Ppp2ca* conditional knockout mouse using the Loxp system to study the function of PP2Acα in the epidermis [10]. We used *Krt14-Cre* transgenic mice to obtain *Ppp2ca* knockout in the epidermis. The Cre recombinase is expressed in *Krt14-Cre* mice under the control of the human keratin 14 (*Krt14*) promoter [11]. It is an effective transgene model for generating conditional knockout mutants to study the function of critical developmental genes in the ectoderm and its derivatives. The findings of this research may be useful in future studies of genetic dermatosis.

## 2. Results

### 2.1. Genotyping and Tissue-Specific Recombination in Ppp2ca^flox/flox^; Krt14-Cre Mice

**Figure 1:**
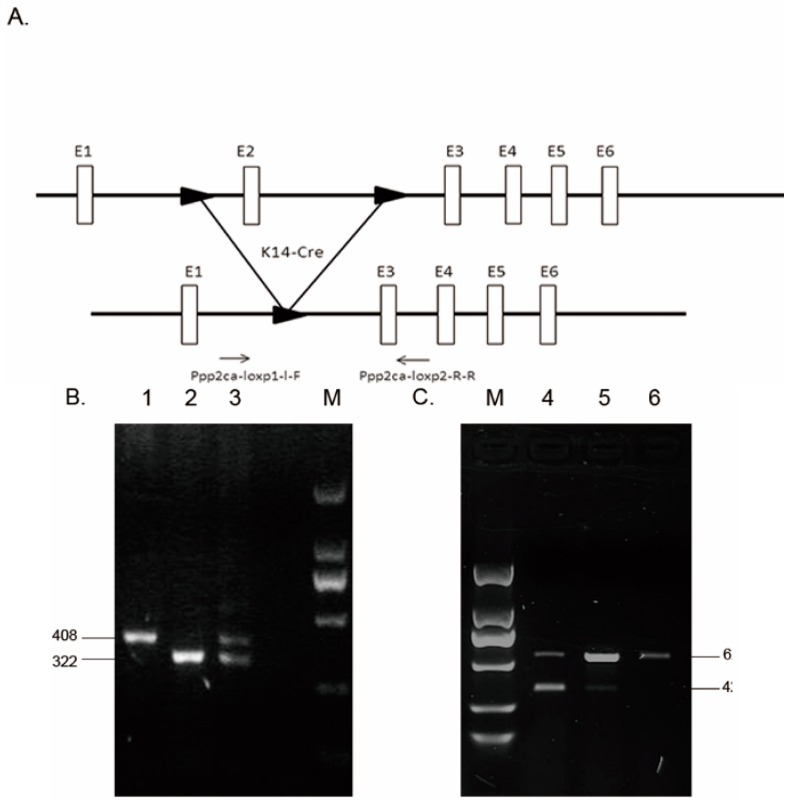

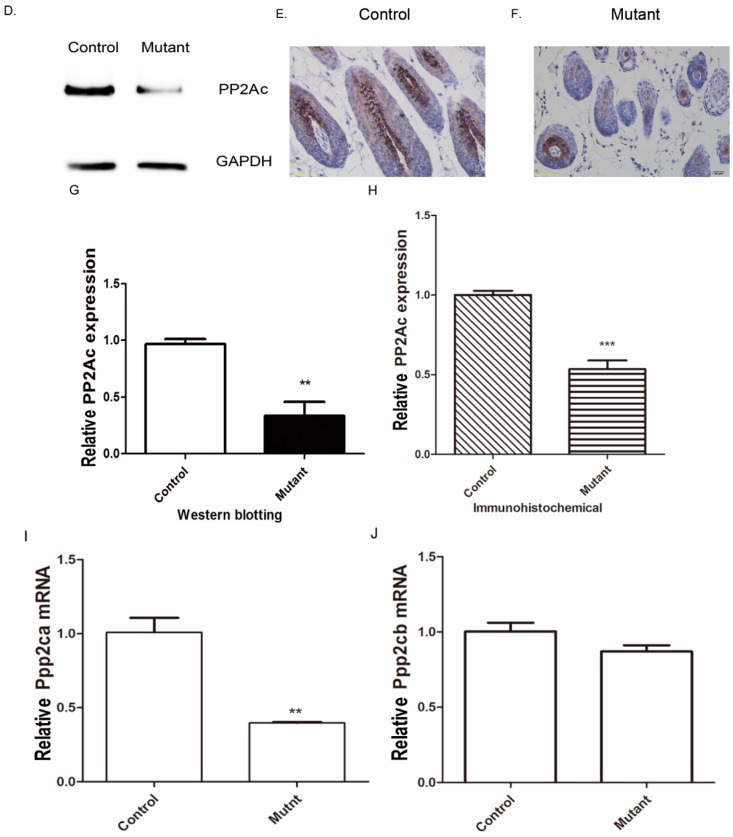
Development of *Ppp2ca* conditional knockout mice. (**A**) The recombination process and the Loxp sites used for knocking out *Ppp2ca*; (**B**) *Ppp2ca^flox/flox^*; *Krt14-Cre* was subjected to PCR analysis. Lanes 1, 2, and 3 represent the wild-type (WT), *Ppp2ca^flox/flox^* and *Ppp2ca^flox/+^*, respectively; (**C**) Back skin of the *Ppp2ca^flox/flox^*; *Krt14-Cre* (lane 4), *Ppp2ca^flox/+^*; *Krt14-Cre* (lane 5) and WT (lane 6) mice were analyzed by reverse transcription PCR. Detection of transcripts shorter than the control mice transcript in mutant mice indicated that the knockout was successful; (**D**) Western blotting with the anti-PP2Ac antibody was used to detect PP2Ac protein expression in the skin of *Ppp2ca^flox/+^*; *Krt14-Cre* and *Ppp2ca^flox/flox^*; *Krt14-Cre* littermates. PP2Ac expression was significantly lower in mutant mice than in controls. The loading control was GAPDH; (**E**,**F**) PP2Ac expression in the hair follicle epithelium of mutant mice was significantly reduced. Scale bars = 20 μm; (**G**,**H**) Statistical analysis indicated that PP2Ac was significant reduced in mutant mice skin; (**I**,**J**) Quantitative real-time PCR assay was carried out to examine the expression levels of *Ppp2ca* and *Ppp2cb* mRNA. *n* = 3 in each group, ** *p* < 0.01, *** *p* < 0.001.

In mice, *Ppp2ca* maps to chromosome 11, consists of seven exons, and encodes a 1930 bp mRNA. We generated conditional *Ppp2ca* knockout mice using the Cre-Loxp recombinase system (Figure 1A) [10]. The generated *Ppp2ca^flox/flox^* mice appeared normal and fertile. *Krt14-Cre* mice were crossed with *Ppp2ca^flox/flox^* mice to obtain *Ppp2ca^flox/flox^*; *Krt14-Cre* mice. These mice were used to study the role of *Ppp2ca* in epithelial tissues (Figure 1B). The *Ppp2ca* transcript was shorter in the *Ppp2ca^flox/flox^*; *Krt14-Cre* mice than in control mice due to the occurrence of the K14-Cre and Loxp recombination (Figure 1C). Next we used anti-PP2Ac antibody to detect the expression of PP2Acα in the epidermis of mice, as shown by the Western blot analysis presented in Figure 1D; the PP2Ac protein expression level was significantly lower in the epidermis of the control group. In addition, the results of the immunohistochemical analysis indicated that the expression of PP2Ac was lower in the mutant than in the control group, as shown in Figure 1E,F. Moreover, the mRNA expression level of *Ppp2ca* was significantly reduced, while *Ppp2cb* showed obvious change (Figure 1G,H). Based on all the above, we successfully generated a epidermal conditional *Ppp2ca*-knockout mouse model by deleting exon 2 of *Ppp2ca*.

### 2.2. Conditional Knockout of Ppp2ca Causes Developmental Disorders

**Figure 2:**
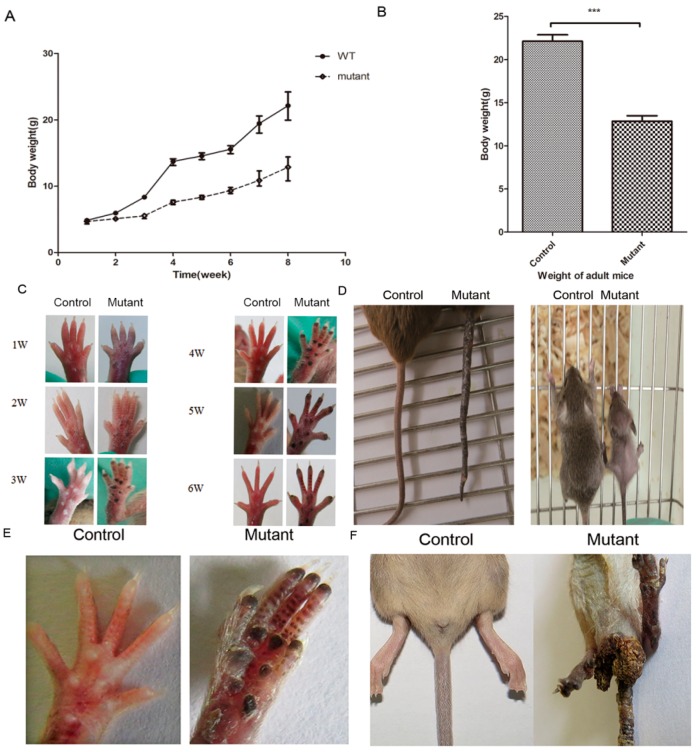
Developmental disorders in epithelial cell–specific *Ppp2ca* knockout mice. (**A**) Change trend curve of the body weight of *Ppp2ca^flox/flox^*; *Krt14-Cre* mouse and littermate control groups. *n* = 6 animals/genotype; (**B**) Body weight of eight-week *Ppp2ca^flox/flox^*; *Krt14-Cre* mouse and littermate control group. *n* = 3 in each group, *** *p* < 0.001; (**C**) Paw pigmentation at different weeks of *Ppp2ca^flox/flox^*; *Krt14-Cre* and littermate control group. Base of the claws in the mutant mice and the control group at one to six weeks; (**D**) Tail of normal (**left**) and mutant (**right**) mice at eight weeks. *Ppp2ca^flox/flox^*; *Krt14-Cre* mice have a smaller body and exhibit hair loss compared to the littermate control group; (**E**) Claws on bottom of normal (**left**) and mutant (**right**) mice at eight weeks; (**F**) *Ppp2ca^flox/flox^*; *Krt14-Cre* mice exhibit anal plug and bowel problems.

The mutant mice gained weight at a significantly slower pace than their normal control littermates (Figure 2A). Adult mutant mice were significant lighter than control mice (Figure 2B). *Ppp2ca^floxflox^*; *Krt14-Cre* mice exhibited visible melanin pigmentation at postnatal day 7, and the blackening of the feet became increasingly apparent (Figure 2C). In addition, the *Ppp2ca^flox/flox^*; *Krt14-Cre* mice also exhibited hair loss (Figure 2D), and the tails of mutant mice had excessive keratinization and melanin deposition (Figure 2D,E). Compared with control mice, the mutant mice were smaller in size (Figure 2D) and had difficulty in excretion (Figure 2F). The anal canal of mutant mice was plugged by feces. The severity of the abnormal phenotypes was age-related, and 15% of the mice exhibiting severe phenotypes died four to six weeks after birth. Our data demonstrate the importance of epithelial *Ppp2ca* signaling in physical development.

### 2.3. Hair Follicle Cycle Is Disrupted in Ppp2ca^flox/flox^; Krt14-Cre Mice

**Figure 3:**
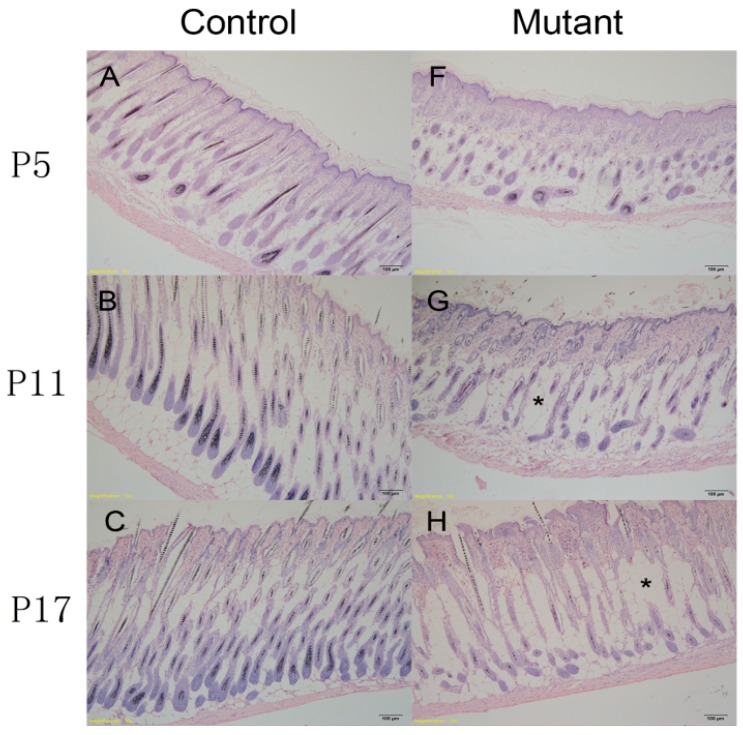

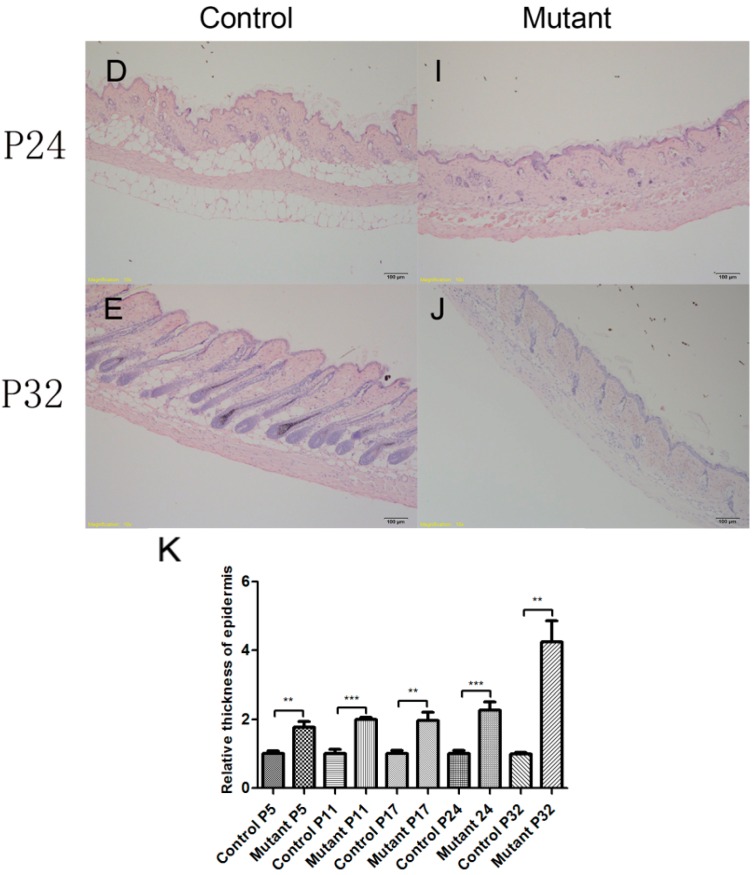
Disruption of the hair follicle cycle in *Ppp2ca^flox/flox^*; *Krt14-Cre* mice. H&E staining of skin tissue sections from the control (**A**–**E**) and mutant (**F**–**J**) at P5 (panels **A** and **F**), P11 (panels **B** and **G**), P17 (panels **C** and **H**), P24 (panels **D** and **I**) and P32 (panels **E** and **J**). At P5, hair follicle development has no obvious difference between the control and mutant groups, which were all at the inducing follicular morphogenesis phase (panels **A** and **F**). The skin of control mice at P11 was at the anagen phase, whereas the follicles of the mutant mice were at a mixed anagen and inducing follicular morphogenesis phase (panels **B** and **G**). At P17, follicles of control mice were at the catagen phase and those of mutant mice were at a mixed anagen and catagen phase (panels **C** and **H**), respectively. At P24, the follicles of the control mice transitioned from the telogen to the anagen phases and the mutant mice were mostly at the catagen phase (panels **D** and **I**), respectively. At P32, the follicles of the control mice entered the full anagen phase and mutant mice remained at the catagen and telogen phases (panels **E** and **J**), respectively. * indicates increased number of fat cells. Scale bars = 100 μm; (**K**) Statistical analysis indicated that relative thickness of mutant mice epidermis was significantly increased compared with the control group at all stage (P5–P32). *n* = 3 in each group, ** *p* < 0.01, *** *p* < 0.001.

In general, hair loss is closely related to the hair cycle [12]. To examine whether the hair loss in our mutant mice (Figure 2D) was related to the hair cycle, skin samples from knockout mice and control littermates at different developmental stages were subjected to histopathological analysis and then compared. Skin samples were obtained from the follicular morphogenesis (P5), anagen (P11), catagen (P17), telogen (P24), and anagen (P32) stages [13]. These mutant mice showed abnormal hair follicle morphogenesis from the first postnatal hair cycle. For example, at P5, hair follicle morphogenesis of the knockout mice was blocked and disorganized, and the hair follicles were of irregular size (Figure 3A,F). At P11, the hair follicles of the mutant mice were in the early anagen phase, whereas those in the control mice were in the fully developed anagen phase. Hair shafts that had adverse differentiation and loss of nuclei were observed in mutant mice (Figure 3B,G). At P17, the dorsal follicles of the control mice were in the catagen phase, whereas the development was delayed in mutant mice, which had some hair follicles that had not yet reached the catagen phase (Figure 3C,H). At P24, the follicles of the control and mutant mice started to enter the anagen phase from the telogen phase (Figure 3D,I). At P32, the skin follicles of control mice entered the new anagen phase, whereas the follicles of the mutant mice appeared not to be under control (Figure 3E,J). These results illustrate that PP2Acα-dependent signaling is essential for hair cycles and, thus, adequate hair development. Additionally, the epidermis was thicker in the knockout mice than in the control group (Figure 3K). As shown in Figure 3G,H, fat cells (*) were increased in the skin of mutant mice.

### 2.4. Ppp2ca Is Required for the Keratinized Cortex and Inner Root Sheath (IRS) in Hair Differentiation

**Figure 4:**
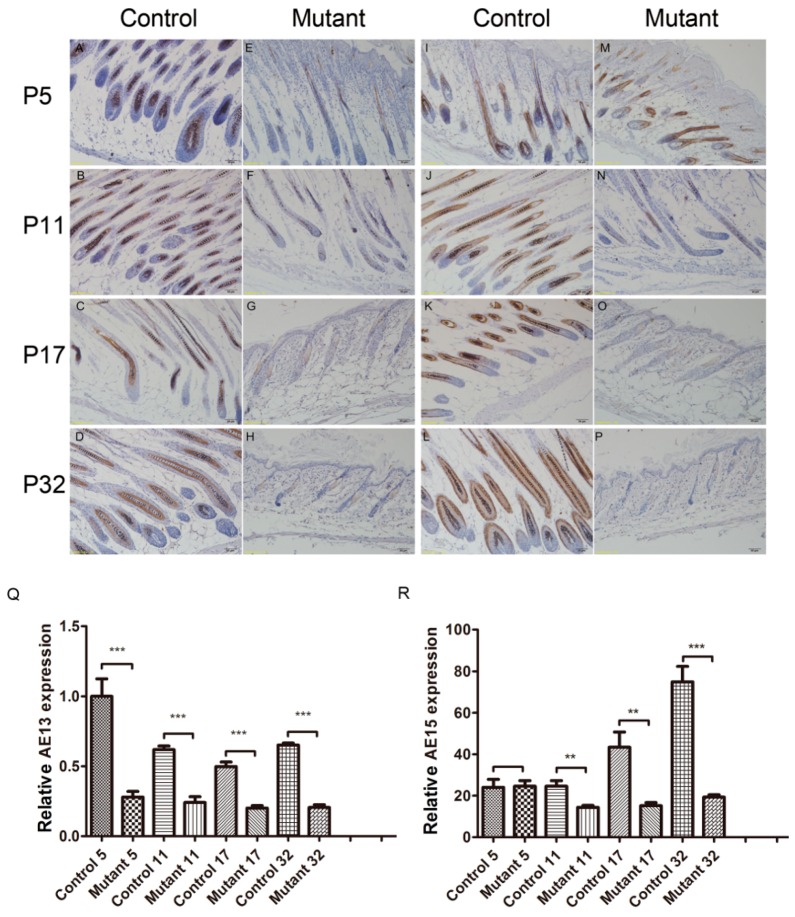
Epithelial PP2Acα signaling is required for the keratinized cortex and IRS in hair differentiation. The antibody to AE13 stains the precortex and cortex of the hair keratin in control mice (panels **A**–**D**) and mutant mice (panels **E**–**H**). The antibody to AE15 stains the IRS and medulla of hair follicles in control mice (panels **I**–**L**) and mutant mice (panels **M**–**P**). Scale bars = 50 μm; (**Q**,**R**) Statistical analysis indicated that AE13 expression in mice skin was significantly reduced in all phases. AE15 expression was significantly reduced in all phases except in the P5 phase. *n* = 3 in each group, ** *p* < 0.01, *** *p* < 0.001.

The normal mouse epidermis contains an interfollicular epidermis with well-defined basal and suprabasal layers, including pilosebaceous units with an outer root sheath, Inner Root Sheath (IRS), hair matrix, and sebaceous glands. Differentiation of hair follicles is closely related to hair loss. A previous study has shown that an anti-AE13 antibody, which targets type I low-sulfur hair shaft cortex keratin in the normal follicle [14], can stain the keratin of the cortex and precortex in hair follicles. The cortex makes up the majority of the hair shaft, and AE15 affects the medulla of the hair shaft and the IRS. Together, antibodies against these two compounds can serve as markers of hair follicle differentiation. Indeed, as shown in Figure 4, AE13 expression was significantly reduced in *Ppp2ca^flox/flox^*; *Krt14-Cre* mice compared to controls as the hair follicles became disorganized (Figure 4A–H,R) at all stages. Staining of AE15 revealed no significant changes in AE15 expression at P5 (Figure 4I–P,S). From P11 onwards, follicular dysplasia decreased and the hair follicles were even lost (Figure 4N) in mutant mice, indicating that the mutant mice have abnormalities in differentiation in the IRS layer and hair shaft angle of the cortex medulla. All the data of this study indicated that the matrix cells failed to differentiate toward the IRS and hair shaft in the follicles of mutant mice. These events might have contributed to the subsequent failure in hair cycling and PP2Acα signaling as an important role in hair differentiation.

### 2.5. Hyperproliferation of Epidermis in Ppp2ca^flox/flox^; Krt14-Cre Mice and AKT Signling Pathway Changed

**Figure 5:**
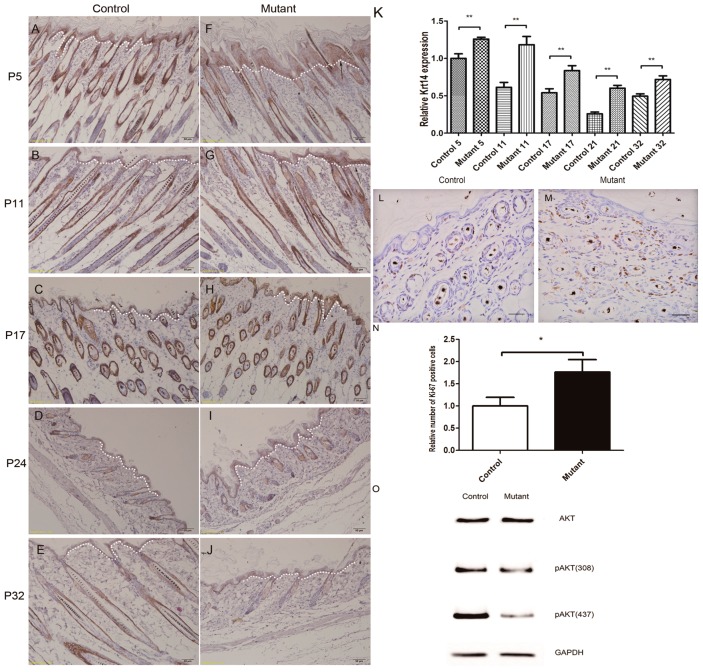
Hyperproliferation of epidermis in *Ppp2ca^flox/flox^*; *Krt14-Cre* mice and AKT signaling pathway changed. An antibody to Krt14 was used to stain tissue sections from the control (**A**–**E**) and mutant (**F**–**J**) dorsal skin at the follicular morphogenesis (P5; panels **A** and **F**), anagen (P11; panels **B** and **G**), catagen (P17; panels **C** and **H**), telogen (P24; panels **D** and **I**), and anagen (P32; panels **E** and **J**) phases. Arrows indicate a conspicuously higher number of Krt14-positive cells in the skin of mutant mice than in the epidermis of the control mice (panels **A**–**J**). All scale bars = 50 μm; (**L**,**M**) Ki67 antibody staining showed hyperproliferation in the epidermis of eight-week mutant mice epidermis compared to control littermates. All scale bars = 20 μm; (**K**,**N**) Statistical analysis indicated that the expression of Krt14 in mice skin was significantly increased at all stage. Statistical analysis indicated that the Ki67 positive cells were increased in mutant mice epidermis. *n* = 3 in each group, * *p* < 0.1, ** *p* < 0.01; (**O**) Western blot analysis of AKT, pAKT (S473), pAKT (T308), from mouse epidermis protein extracts from the knockout group and the control group. The Krt14-positive cell area was indicated by white dotted lines.

Keratin *GPx4* gene knockout mice were found to have significant epidermal hyperplasia, and the *Krt14*-positive cells in the epidermal layer increased significantly [15]. In order to further investigate the follicular and interfollicular epidermal proliferation, immunostaining was performed using keratin 14 and Ki-67 antibodies. Our study revealed that the number of Krt14-positive cells was increased in the epidermis of *Ppp2ca* conditional knockout mice (Figure 5A–K). Ki67 results indicated that hyperproliferation of basal cells occurred in the mutant mice epidermis (Figure 5L–N). From the above results, we can conclude that epidermal hyperplasia of the epidermis in mutated mice, which induced epidermal thickness in mutant mice, was increased (Figure 5K). We also found no difference regarding the total amount of AKT expression and an obvious decrease of AKT-phosphorylation in mutant mice (Figure 5O).

## 3. Discussion

The *Ppp2ca* gene encodes the α isoform of the PP2A catalytic subunit (PP2Acα). As a tumor suppressor gene, *Ppp2ca* is a hotspot for tumor treatment [16]. Besides, *Ppp2ca* is also known to be associated with tauopathies, such as Alzheimer’s disease and others [17,18]. Previously, we have successfully generated *Ppp2ca* conditional knockout mice in testes using *DDX4-Cre* and *Ppp2ca^flox/flox^* mice, which have the exon 2 of *Ppp2ca* deleted. Knocking out *Ppp2ca* exon 2 causes changes in testicular morphology and infertility [10]. 

*Krt14-Cre* mice have been widely used in skin disease research because of the tissue-specific expression of the Krt14 promoter [19]. Therefore, we used *Krt14-Cre* and *Ppp2ca^flox/flox^* mice to conditionally knock out *Ppp2ca* in the epidermis and examine the impacts on epidermal development. In mutant mice skin, expression of the PP2Ac protein did not entirely disappear. Two reasons can account for this. First, PP2Ac consists of PP2Acα and PP2Acβ, and we just knocked out PP2Acα; however, while PP2Acβ mRNA expression was not significantly changed (Figure 1J), it can be detected by the anti-PP2Ac. Second, in *Krt14-Cre* expression in keratinocytes of the epidermis, a certain degree of expression of PP2Ac from other types of cells in the epidermis can be detected.

PP2A was reported to negatively regulate the AKT pathway [20]. However, in our study, phosphorylated AKT was decreased in the mutant mice skin, which indicated knocking out *Ppp2ca* in mice skin leads to the promotion of the AKT pathway. Apart from PP2A, phosphorylation of AKT was found to be regulated by various pathways [21,22]. This indicated that PP2A positively regulates AKT through other signaling pathways rather than by direct negative regulation of the AKT signaling pathway, but the details of the underlying mechanism remain unclear. Activation of AKT leads to β-catenin stabilization and its nuclear accumulation, which can positively regulate the Wnt pathway [23]. Wnt signaling is involved in the pathogenesis of many skin diseases and epidermis development. In fibrotic diseases, the canonical Wnt signaling pathway has been established as an important mediator of sustained fibroblast activation [24]. In addition, during hair follicle induction and morphogenesis, the Wnt pathway also plays a crucial role and is considered to be the master regulator [25]. We speculate that PP2A promotes the Wnt signaling pathway in hair follicles and the inactivation of *Ppp2ca* in mutant mice can affect Wnt signaling and induce hair follicle dysplasia. In our study, the increased hyperplasia in mutant mice suggests that the effects could be due to knocking out an inhibitor of Wnt signaling, not an activator [26]. PP2A can either activate or inhibit Wnt signaling, depending on the bound “B” regulatory subunit [27]. Each B subunit family has several isoforms which can mutually exclusively bind to the AC dimer [28]. The B55a subunit directly binds to the β-catenin destruction complex, where PP2A dephosphorylates β-catenin and induces its degradation [29]. Overexpression of B56 family members inhibits Wnt/β-catenin signaling during Xenopus embryonic development [30,31]. We hypothesize that knocking out *Ppp2ca* induces changes in the B regulatory subunit, which can affect the role of PP2A in the Wnt signaling pathway, and the increased hyperplasia was caused through the negative regulation of the Wnt signaling pathway by PP2A. The different regulatory roles of PP2A in different cells may be due to its diverse regulatory subunit family.

Previous studies have indicated that *Krt14-Cre* mice have high Cre activity in the epidermis, hair follicles, oral epithelium and developing teeth, and low Cre activity in the esophagus and forestomach at embryonic day 14.5 [32,33]. In this study, the oral epithelium and teeth of *Ppp2ca* conditional knockout mice model were normal. Mutant mice were obstructed by stool (Figure 2F). All the mice were raised under specific-pathogen-free (SPF) conditions, so we exclude the diet factor and environmental factors on defecation. We conjecture that expression of Cre in the digestive system and bowel problems causing malnutrition are possible important reasons for the observed physical development. We suspect that ectopic expression of different levels of Krt-14 Cre in digestive system and different physiques may have caused of the death of 15% of the mutant mice three to four weeks after birth.

The skin-specific *Ppp2ca* knockout mice appeared stunted, and exhibited hyperproliferation and melanin deposition of at the bottom of the claws and tail. The molecular mechanisms related to these abnormal phenotypes warrant further research. In conclusion, our study demonstrates that PP2Acα plays important roles in both hair follicle and skin development. Our transgene model may provide some novel clues for understanding the molecular mechanisms of genetic skin diseases and identifying new targets for treatment. In addition, the *Ppp2ca^flox/flox^* mice in this study can also serve as a useful tool to study the role of *Ppp2ca* in other developmental processes and diseases.

## 4. Materials and Methods

### 4.1. Animals

All animal protocols were approved by the Animal Care and Use Committee of the Model Animal Research Center of Nanjing Medical University. This study was approved by the Institutional Animal Care and Use Committee of Nanjing Medical University (Number: NAJMU-IACRCUC-20100601001). All mice were fed by standard diet and housed in specific-pathogen-free barrier facilities.

### 4.2. Generation of Ppp2ca^flox/flox^; Krt14-Cre Mice

To achieve the specific deletion of the *Ppp2ca* gene in the epidermis, we crossed *Ppp2ca^flox/flox^* mice with the *Krt14-Cre* transgenic mice (keratin cell-specific Cre line, J004782, Jackson Laboratory, Bar Harbor, ME, USA). *Ppp2ca^flox/+^*; *Krt14-Cre* mice were genotyped by PCR (Cre-F/R; Ppp2ca-loxp1-L-F/Ppp2ca-loxp2-R-R). Subsequently, we bred the *Ppp2ca^flox/+^*; *Krt14-Cre* mice with the *Ppp2ca^flox/flox^* mice to obtain *Krt14-Cre*; *Ppp2ca^flox/flox^* mice. In brief, *Cre*-induced recombination would delete exon 2 of the *Ppp2ca* gene and result in a frameshift mutation that eliminates *Ppp2ca* expression. PCR reactions were performed in PCR buffer (25 mL) containing 0.2 mM dNTP (Promega, Madison, WI, USA), forward and reverse primer (0.2 mM each), template DNA (1 mL), DMSO (5%; *v*/*v*, Merck, Darmstadt, Germany), MgCl_2_ (4 mM), and 0.25 mL AmpliTaq GoldTM (Roche, Penzberg, Germany). Amplification was performed as follows: initial denaturation, 94 °C for 5 min; 35 PCR cycles, 94 °C for 30 s, 55 °C for 30 s, and 72 °C for 30 s; and final extension, at 72 °C for 10 min. Amplification of the *Krt14-Cre* transgene generated a 374 bp DNA fragment. The PCR specific primers sequences are listed below:

Ppp2ca-loxp1-L-F: 5′-AATAATGCGGCCGCAACCCCAACAACAACCACA-3′
Ppp2ca-loxp2-R-R: 5′-AATAATGTCGACACCATCTACTCTAAACTCTCCACTT-3′
Cre-F: 5′-TTGCCTGCATTACCGGTCGATGC-3′
Cre-R: 5′-TTGCACGTTCACCGGCATCAACG-3′

### 4.3. RNA Preparation and Real-Time PCR

Total RNA was extracted from skin tissue using RNAiso Plus according to the manufacturer’s protocol (TaKaRa, Tokyo, Japan). A PrimeScript^TM^ reverse transcription reagent kit with gDNA Eraser (TaKaRa) was used with random primers (Invitrogen, Carlsbad, CA, USA) to obtain cDNA. The cDNA of *Ppp2ca* was detected by primers Mpp2ca-P5F and Mpp2ca-P5R. The PCR products were 623 bp (control) and 423 bp (knockout). The PCR specific primers sequences are listed below:

Mpp2ca-P5F: 5′-GGTCAAGAGCCTCTGCGAGAA-3′
Mpp2ca-P5R1: 5′-CCGGTCATGGCACCAGTTAT-3′

Real-time quantitative PCR was conducted using GoTaq^®^ qPCR Master Mix (SYBR Green) (Promega) on a Roche LightCycler480 system (Roche Applied Science, Indianapolis, IN, USA). The qPCR procedure was as below: 94 °C for 2 min; 40 PCR cycles, 95 °C for 15 s, 55 °C for 15 s and 68 °C for 25 s. *Ppp2ca* and *Ppp2cb* mRNA relative expression normalized to β-actin expression and calculated using the 2^−∆∆*C*t^ method with efficiency correlation. The sequences of the specific primers used for PCR are listed below:

Ppp2ca-qf: 5′-ATGGACGAGAAGTTGTTCACC-3′
Ppp2ca-qr: 5′-CAGTGACTGGACATCGAACCT-3′
Ppp2cb-qf: 5′-GAGGGTACTACTCTGTGGAGAC-3′
Ppp2cb-qr: 5′-CCGGCTTTCGTGATTTCCT-3′
M-β-actin-S: 5′-GTGACGTTGACATCCGTAAAGA-3′
M-β-actin-A: 5′-GTAACAGTCCGCCTAGAAGCAC-3′

### 4.4. Western Blotting

Protein extraction was quantified according to standard procedures, and proteins were subjected to electrophoretic separation (20 μg protein/lane) and electroblotting onto PVDF membranes. Skin was homogenized in hypotonic solution. A mix of protease inhibitors (Roche, Penzberg, Germany) were also added to the buffer. The following antibodies are listed below: rabbit monoclonal PP2Ac antibody (1:1500, Cell Signaling Technology, Inc., Beverly, MA, USA), rabbit antibody AKT (1:1500, Cell signaling, Inc., Beverly, MA, USA), rabbit monoclonal antibody pAKT (S473) (1:2000, Cell signaling, Inc., Beverly, MA, USA), and rabbit monoclonal antibody pAKT(T308) (1:1000, Cell signaling, Inc., Beverly, MA, USA), rabbit monoclonal GAPDH antibody (1:1000, Sigma-Aldrich, St. Louis, MO, USA), and goat anti-rabbit IgG-HRP antibody (1:2000, Sigma-Aldrich).

### 4.5. Histological Analysis

Skin samples were fixed in Bouin’s fixative solution overnight at room temperature. On the next day, tissues were dehydrated through an ethanol series and cleared in xylene. Tissues were embedded in paraffin, and cut into 5 μm sections. For histological analysis, sections were stained with hematoxylin and eosin (H&E).

### 4.6. Immunohistochemistry

Indirect immunofluorescence staining was carried out on paraffin sections using the following primary antibodies: rabbit anti-PP2Ac (1:100, Cell Signaling); mouse anti-cytokeratin-14 (1:50, Santa Cruz, Dallas, CA, USA); mouse anti-pan-cytokeratin (1:50, AE13, Santa Cruz, Dallas, CA, USA); mouse anti-trichohyalin antibody (1:100,AE15, Abcam, Cambridge, UK); Rabbit anti-Ki67 (1:100, Abcam, Cambridge, UK); secondary antibody kit: Rabbit SP detection kits (KIT-9706) (Maxim, FuZhou, China), mouse SP detection kits(KIT-9701) (Maxim). Quantification of fluorescence was performed by a blinded observer using the ImageJ software (National Institute of Mental Health, Bethesda, MD, USA) and depicted as percent of relative expression.

### 4.7. Statistical Analysis

Statistical analysis was performed using the PRISM Graph pad 5.01 software (Graphpad Inc., Carlsbad, CA, USA). *p* < 0.05 was considered significant.
